# Selected tools to visualize membrane interactions

**DOI:** 10.1007/s00249-021-01516-6

**Published:** 2021-03-31

**Authors:** Tobias Grothe, Julia Nowak, Reinhard Jahn, Peter Jomo Walla

**Affiliations:** 1 Laboratory of Neurobiology, Max-Planck-Institute for Biophysical Chemistry, 37077 Göttingen, Germany; 2 Department of Biophysical Chemistry, Institute for Physical and Theoretical Chemistry, University of Braunschweig, 38106 Braunschweig, Germany

**Keywords:** Fluorescence cross-correlation spectroscopy, Fluorescence lifetime analysis, Liposomes, Membrane fusion intermediates, Synaptic proteins

## Abstract

In the past decade, we developed various fluorescence-based methods for monitoring membrane fusion, membrane docking, distances between membranes, and membrane curvature. These tools were mainly developed using liposomes as model systems, which allows for the dissection of specific interactions mediated by, for example, fusion proteins. Here, we provide an overview of these methods, including two-photon fluorescence cross-correlation spectroscopy and intramembrane Förster energy transfer, with asymmetric labelling of inner and outer membrane leaflets and the calibrated use of transmembrane energy transfer to determine membrane distances below 10 nm. We discuss their application range and their limitations using examples from our work on protein-mediated vesicle docking and fusion.

## Introduction

Membranes are a pivotal part of all biological organisms. In an aqueous environment, they form amphiphilic bilayers that are not permeable for hydrophilic molecules. They not only provide a barrier that separates the cellular reaction space from the outside world, but they also allow for the formation of segregated compartments in eukaryotic cells. In all organisms, membranes are continuously remodeled by fusion and fission. Both processes are tightly regulated and governed by specific protein machineries that guide the membranes through metastable non-bilayer intermediates.

Fusion of biological membranes can either occur in the extracellular space, for instance, fertilisation [reviewed e.g. in (Chen and Olson 2005)] or cell invasion by enveloped viruses [reviewed e.g. in (Harrison 2015)], or in the intracellular space, for instance during fusion of mitochondria or of trafficking vesicles with other membranes such as the fusion of synaptic vesicles with the presynaptic membrane (Chen and Scheller 2001; Chernomordik and Kozlov 2008; Sudhof and Rothman 2009). Although the proteins mediating membrane fusion are structurally diverse and evolved independently, it appears that membrane fusion generally follows a common pathway involving structurally similar intermediates (Hernandez and Podbilewicz 2017): First, the to-fuse membranes are brought into proximity. Next, the membranes are connected by proteins anchored in both participating membranes. Frequently, two steps are distinguished: a first step, in which the membranes are still several nm apart and connected by extended proteins (also referred to as “tethering”), and a second step, in which the membranes are tightly apposed and less than 1–2 nm apart (also referred to as “docking”). Next, major structural rearrangements occur in the fusion proteins that follow a downhill energy gradient and draw both membranes together. As a result, the hydration barrier is overcome, resulting in the formation of a fusion stalk or a hemifusion intermediate in which only the proximal lipid leaflets are fused, followed by rupture of the hemifusion diaphragm and full fusion (Diao et al. 2012).

The use of model systems where isolated membrane proteins were reconstituted into artificial liposomes was instrumental for our present understanding of membrane fusion as it allows for precise control of the environment and the involved proteins (Marsden et al. 2011). However, the structural characterization of fusion intermediates is impeded by the fact that membranes are highly dynamic, consist of assemblies of thousands of amphiphilic molecules and undergo complex phase transitions that cannot be easily visualized by conventional methods known from the biosciences (Brunger et al. 2015). For instance, membrane docking, hemifusion, fusion, distances between membranes, membrane curvature, and local shape fluctuation are difficult to monitor at the nanometer scale. To get information about these features under dynamic, ambient and physiological conditions, one cannot exclusively rely on information extracted from images but rather needs complementary approaches.

In our review, we discuss selected approaches to gain more insight into these membrane-related features. We cover methods differentiating docked from fused membranes in freely diffusing liposome model systems (Cypionka et al. 2009; Hernandez et al. 2012; Vennekate et al. 2012), methods providing insights about hemi-fused membranes under the same conditions (Lin et al. 2016), and methods determining membrane distances at a length scale below 10 nm (Lin et al. 2014). There are additional methods that visualize membrane orientation and curvature in entire cells (Hafi et al. 2014, 2016), or membrane growth and morphology in living cells (Chen et al. 2015). While all of these methods add much to the overall understanding of membranes, their mechanisms and role in essential processes, here, we will focus on selected methods that allow to observe elementary features of interactions between membranes and membrane–protein interactions in a controlled manner using liposome model systems (Brunger et al. 2015; Rigaud and Lévy 2003).

## Differentiation between docking and fusion using two-photon fluorescence cross-correlation spectroscopy and intramembrane Förster energy transfer

As discussed above, fusion proceeds via consecutive steps involving tethering, docking, hemifusion, and full fusion. To understand how individual proteins participating in fusion contribute to these transition states, it is necessary to use methods that allow for quantification of the various intermediates. In our work, we have used liposomes as model systems since they allow for controlling the protein and lipid composition of the membrane as well as membrane curvature and tension. In most cases, two different types of liposomes are prepared that model, for instance, the synaptic vesicle and presynaptic membrane, respectively, and that can be differentially labelled with different fluorescence tags. When working with such liposome model systems one can generally differentiate between assays with freely diffusing liposomes (Struck et al. 1981; Weber et al. 1998) and others that comprise at least one liposome species attached to a solid support such as a planar surface (see, e.g., Diao et al. 2010; Kyoung et al. 2013; Yoon et al. 2006)). Alternatively, silica spheres have been coated with lipid bilayers (see, e.g., (Oelkers et al. 2016)). Approaches involving surface-immobilized liposomes have the advantage that interaction partners of individual liposomes can be monitored over extended periods. On the other hand, immobilization might introduce liposome deformation and increase curvature stress which may alter the lifetime of the intermediate steps of the fusion process. Additionally, non-specific adhesion may comprise the detection of those features (Witkowska and Jahn 2017). In contrast, methods in which all liposomes are kept in suspension allow for avoiding the discussed concerns but require methods other than imaging.

In the three sections of this article, we focus on the latter systems, involving only interactions between membranes freely diffusing in an aqueous environment. How can one distinguish between free, tethered or docked and fused membranes when working with small liposomes in solution? We addressed this question in an earlier paper that describes the development of a liposome docking and fusion assay based on two photon, two colour fluorescence correlation spectroscopy (FCCS) as well as intramembrane Förster resonance energy transfer (FRET) monitored by fluorescence lifetime (Cypionka et al. 2009). In the following we will describe the functioning of this assay and why we selected this particular approach.

**Fig. 1:**
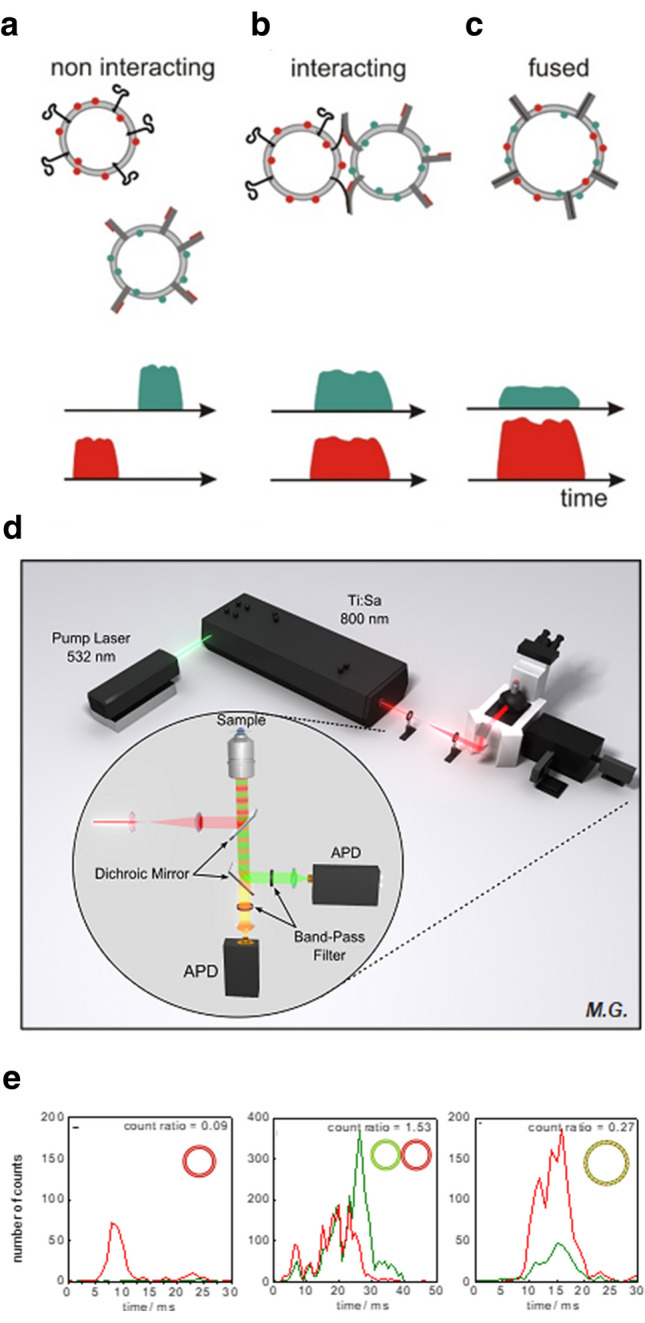
Observation and differentiation of membrane fusion intermediates with a two-photon flluorescence microscope. **a**–**c** Cartoons of the freely diffusing liposome model system at different states of the membrane fusion process and the characteristic fluorescence signals of single liposomes at these states. Two populations of red or green tagged liposomes, if non-interacting (**a**), show temporally independent fluorescence peaks, while, in case of docked/tethered (**b**) and fused (**c**) liposomes, fluorescence peaks of both colors are detected simultaneously. Additionally, fused (**c**) liposomes show an increase in the red fluorescence and a decrease in the green fluorescence. **d** Representation of the two-photon fluorescence microscope. A Ti:Sapphire laser provides high-intensity pulsed light at 800 nm for excitation of the fluorescent tags. The fluorescence light of both tags is collected by the same objective and separated from the excitation light by a first dichroic mirror. The signals are split into red and green by a second dichroic mirror, refined with a corresponding band pass filter and focused onto an avalanche photo diode (APD) for detection. **e** Examples for real fluorescence signals of the states of membrane fusion shown in (**a-c**). Reprinted with permission from Matthias Grundwald and (Cypionka et al. 2009)

In Fig. 1 the principle of this assay is visualized. At least two different populations of liposomes are used. To give an example, one population may be composed of a lipid mixture corresponding to the lipid composition of synaptic vesicles whereas the other may be composed of a lipid mixture resembling the assumed lipid composition of the presynaptic membrane at the synaptic cleft of neuronal cells. Both contain a small amount (on the order of a few percent) of lipids tagged with different fluorescence dyes, respectively (Fig. 1a–c). In our case, we often use Oregon green as well as Texas red for reasons that will be explained in more detail below. In addition, the liposomes may contain distinct synaptic proteins. For example, when modelling neuronal exocytosis, liposomes corresponding to synaptic vesicles may contain synaptotagmin 1, the protein responsible for triggering neurotransmitter release (Park et al. 2015; Vennekate et al. 2012) and one of the SNARE proteins required for fusion, whereas liposomes corresponding to the presynaptic plasma membrane contain complementary sets of SNARE proteins (Hernandez et al. 2012; Yavuz et al. 2018).

Then, these liposomes are observed in a confocal, two-photon fluorescence microscope that contains a pulsed two-photon laser source with excitation wavelengths in the infrared spectral range (typically between 750 and 1100 nm) and detectors that are able to detect the fluorescence decay observed after pulsed excitation with ps-time resolution (Fig. 1d). When two different liposome populations are used that are tagged with two different fluorescence labels (e.g. red and green), two different of such detectors are needed. The detector records the temporal fluorescence signal when red or green labelled liposomes diffusively pass the focal two-photon excitation volume of about 1 fL in size and 250–500 nm in diameter. What type of signals are recorded from diffusing liposomes that are free (Fig. 1a), that are docked or tethered (Fig. 1b), or that are fused (Fig. 1c)? In principle, the primary information is already contained in the schematic signals shown in Fig. 1a–c. When the liposomes do not interact, the signals observed in the donor and acceptor channel are temporally uncorrelated (red and green in Fig. 1a). For docked as well as fused liposomes the signals are temporally correlated (Fig. 1b, c). Signals from docked and fused liposomes are additionally differentiated by intramembrane FRET between the two dyes being at close distance in fused membranes: the fluorescence intensity of the donor dyes (green) decreases while the fluorescence intensity of the acceptor dyes (red) increases, due to energy transfer from the donors to the acceptors (Struck et al. 1981; Weber et al. 1998).

**Fig. 2:**
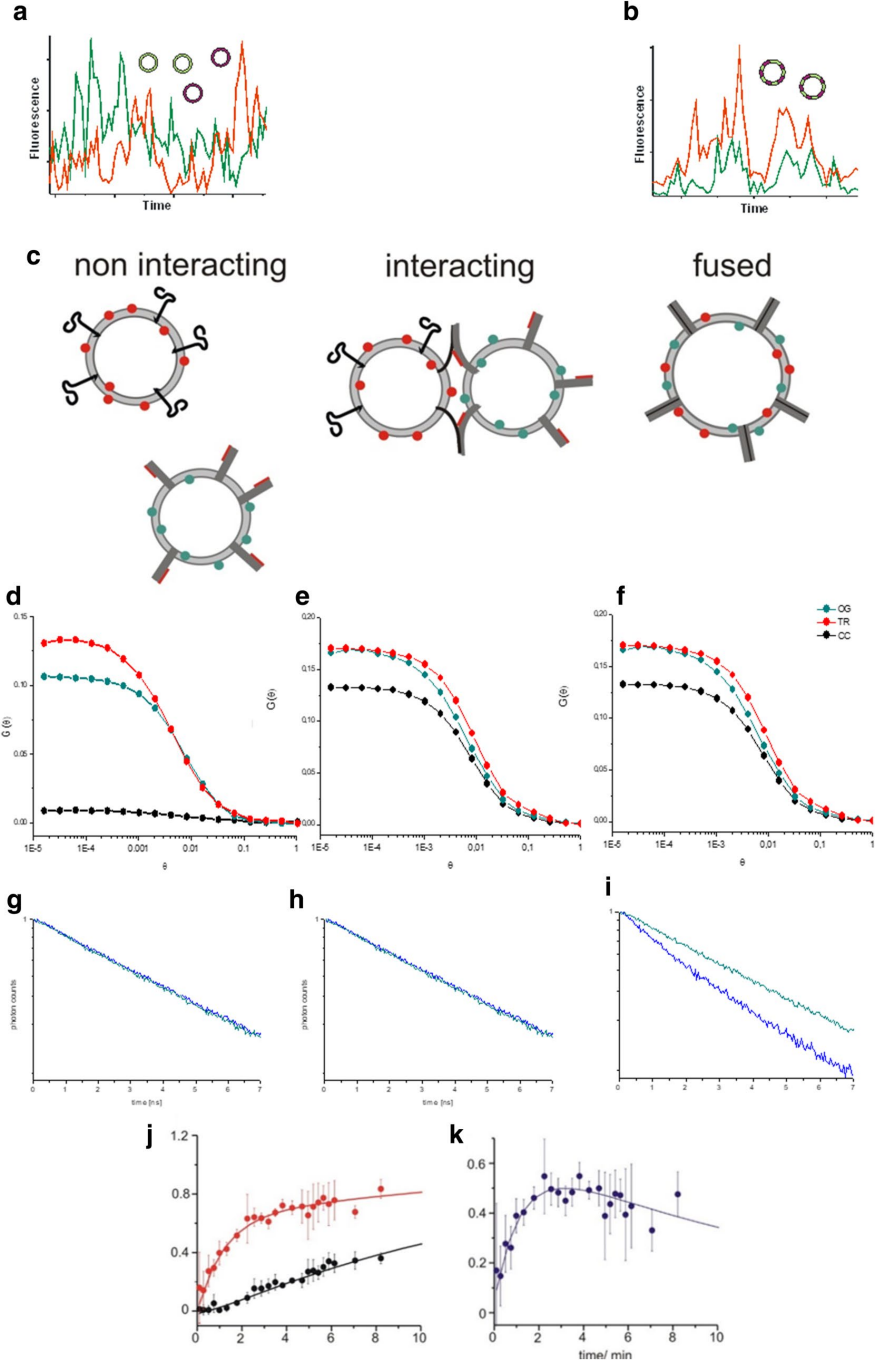
Combination of FCCS and fluorescence lifetime analysis to quantify non-interacting, docked and fused liposomes in a mixture. **a** FCCS signal of multiple differently tagged liposomes. **b** FCCS signal of multiple double-tagged liposomes. **c** Cartoon of non-interacting, interacting and fused liposomes. **d**–**f** Representative autocorrelation and cross-correlation curves for tagged liposomes at different fusion states. The autocorrelation curves are shown in red and green for red and green labelling, respectively. While non-interacting liposomes (**d**) show a cross-correlation amplitude close to zero (black), docked (**e**) and fused (**f**) liposomes show a cross-correlation amplitude close to that of the autocorrelation curves in a 1:1 mixture. **g**–**i** Representative normalised fluorescence decay curves of the FRET donor at different fusion states. Only if the liposomes are fused, (**i**) a significant acceleration of the fluorescence decay is observed. **j** Temporal evolution of the relative cross-correlation and lifetime decrement in a liposome fusion assay driven by isolated neuronal SNARE proteins (Cypionka et al. 2009). The relative cross-correlation (red) represents both, docked and fused liposome populations, while the lifetime decrement (black) represents only the fused liposome population. A subtraction of the latter from the relative cross-correlation results in the representation of only docked liposomes (**k**). Reprinted with permission by (Cypionka et al. 2009; Zwilling et al. 2007)

In principle, counting these signals from individual liposomes or pairs diffusing through the confocal two-photon excitation volume allows for calculating the percentage of free, docked or fused liposomes. Figure 1e shows signals of such single liposome or single liposome pair transits. However, obtaining enough data from single liposome detection is very time consuming and often does not allow for monitoring the temporal changes in the entire population of free, tethered/docked and fused liposomes during an experiment. Therefore, methods are required which provide the same information for many liposomes simultaneously within the detection volume. This can be achieved by two-photon FCCS and FRET-fluorescence lifetime analysis (Fig. 2).

Let us first take a look at the FCCS signals observed for concentrations of significantly more than one liposome in the two-photon excitation volume. Fluorescence cross-correlation is a quantitative measure for the fraction of signals from the red and green fluorescence dyes (“channels”) that are observed together in time. In a sample containing liposome populations that are completely independent, i.e. no interaction is present, the fluctuations of the fluorescence signals of the two different populations (red and green) are completely uncorrelated (Fig. 2a) resulting in cross-correlation curves with an amplitude close to zero (black in Fig. 2e). However, the observed signal fluctuations in the red and green channel are fully correlated, if, in a 1:1 mixture, each red liposome is connected to one green liposome, either by tethering/docking or fusion (Fig. 2b). Consequently the cross-correlation signal nearly matches 100% of that of the correlation signals of the red and green channel alone (Fig. 2e, f). One important advantage of the statistical correlation analysis is that the amplitudes of the cross- as well as auto correlation curves (red and green in Fig. 2d–f) are independent of the actual detected fluorescence brightness of the individual liposomes. If only 50% of the liposomes would be tethered or fused, the cross-correlation amplitude would be 50% of that of the two auto correlation amplitudes, regardless of the fluorescence brightness of each individual liposome and the detection efficiencies of the red or green detectors. In addition, the amplitude of the red and green auto correlation curves give direct information about the relative composition of the mixture of liposomes in cases when there is no 1:1 ratio, again regardless of the individual fluorescence brightness and detection efficiencies of the red or green detector. Therefore, in summary, fluorescence correlation spectroscopy provides very robust information about the relative number of red or green tagged liposomes as well as the percentage of liposomes double tagged due to tethering/docking or fusion.

One advantage of the use of two-photon excitation for this analysis is that pairs of two different fluorescence tags can be selected that can effectively be excited by a single two-photon excitation wavelength in the infrared. In case of Oregon Green and Texas Red, for example, both tags can be equally well excited by two-photon wavelengths at around 800 nm (Cypionka et al. 2009). The identity of the excitation beam for both tags as well as the focal excitation volume, intrinsically restricted to a volume of about 250–500 nm in diameter, ensures 100% overlap of all signals observed from any double labelled liposome (Schwille and Heinze 2001). Such a 100% overlap is necessary for this approach, as an overlap of only 50%, for instance, would result in cross-correlation signals that are also only 50% of the real cross-correlation. Even if one of the detectors (e.g. green) is aligned in a way that it collects less photons than the other detector (e.g. red), the correlation analysis would still result in 100% cross-correlation for a sample of 100% double-tagged liposomes. The cross correlation analysis does not depend on the absolute brightness in the detection—only 100% overlap in the excited volume is necessary. Thus, in two-photon correlation analysis no demanding alignment is necessary to precisely overlap excitation volumes contrary to one-photon excitation with two different beams. In addition, demanding alignment of confocal pinholes is not necessary in the detection paths as the emission observed after two-photon excitation intrinsically originates only from the focal region, in contrast to one photon excitation approaches. Another technical advantage is the requirement of only one dichroic mirror in the confocal set-up separating excitation wavelengths in the infrared (> 750 nm) from the fluorescence tags emission in the visible spectral regions (< 750 nm). Thus, no change of the dichroic mirror and subsequent realignment of the system is necessary when using different fluorescence dyes—the two-photon excitation will be always in the infrared spectral region and the fluorescence detection will be always in the visible spectral region.

While the cross-correlational analysis allows for providing information on the percentage of single- as well as double-tagged liposomes (Fig. 2d–f) it is not able to differentiate tethered/docked liposomes (Fig. 2e) from fused liposomes (Fig. 2f), as both particles contain both dyes. To provide quantitative information of the percentage of the tethered/docked liposomes as well as fused liposomes we take advantage of the fact that effective FRET can occur between Oregon Green and Texas Red when the dyes are less than few nm apart. This is only true for the entire dye population when the dyes are present in the same (fused) membrane (van den Bogaart et al. 2010). Thus, in fused membranes Oregon Green acts as a donor for effective energy transfer to Texas Red and as mentioned above, the Oregon Green fluorescence is significantly depleted while the Texas Red fluorescence is significantly increased in fused membranes. One approach to detect this is a simple comparison of relative changes in the intensity of green and red fluorescence, for example, of individual liposomes passing by the two-photon detection volume. However, simultaneous observation of the fluorescence intensities of larger numbers of red/green labelled liposome mixtures in the focal volume is severely hampered by the large intensity fluctuations intrinsic to correlational analysis and the variations of the donor brightness in free, docked/tethered and fused liposomes that can hardly be dissected.

A more robust read-out than the fluorescence intensity for the extent of energy transfer is the reduction of the lifetime of the donors’ excited state (Zwilling et al. 2007). The lifetime of the excited state of the donor can easily be determined by measuring the fluorescence decay rate of the donor fluorescence after pulsed excitation. In two-photon microscopes, pulsed excitation is intrinsically present. The great advantage of detecting FRET via the excited state lifetime of the donors is that the time constant of the donors fluorescence decay is independent of the fluorescence intensity. Even if the intensity fluctuates due to particles diffusing in and out of the detection volume, the decay itself remains the same. Thus, detecting the fluorescence decay of the donor is a very robust read-out for the total percentage of membranes with donor tags (e.g. Oregon green) fused with membranes tagged with acceptors (e.g. Texas red). If, for example, a sample contains exclusively donor tagged liposomes that are not fused with acceptor tagged liposomes, the measured donor fluorescence decay corresponds to the value observed in the absence of any acceptor tag (e.g. ~ 3.7 ns for Oregon Green, Fig. 2g, h). If a sample contains exclusively fused liposomes, a significantly lower value is observed (e.g. ~ 3.4 ns, Fig. 2i), depending on the concentrations of donor and acceptors in the liposome membranes. As the lifetime can be determined with very high accuracy (typically on the order of about 0.01–0.05 ns) within a couple of seconds this difference can be determined with high significance. In contrast to the FCCS data, however, docked liposomes have a very similar fluorescence decay as free liposomes as at most intermembrane energy transfer can only occur at the fraction of the docked liposomes surface that are close together. Thus, the additional information on the donor fluorescence lifetime allows to distinguish docked/tethered liposomes from fused liposomes. If only 50% of the liposomes are fused, only half of the membranes are mixed and therefore the detected lifetime will be half way between above values (~ 3.55 ns). Of course, this is an oversimplification since such a mixture shows a multiexponential fluorescence decay comprised of two components with individual decay times of ~ 3.7 and ~ 3.4 ns, respectively, with the percentage of fused vesicles being reflected in the ratio of the amplitudes of these two components. However, while the average time constant of a decay can be determined with high accuracy, it is less robust to determine the individual amplitudes, particularly when the two time constants are close to each other. Therefore, the more robust read-out is the average lifetime of e.g. 3.55 ns in case of a 50/50% mixture (Zwilling et al. 2007).

The big advantage of combining the two-photon FCCS and FRET assays is that both read-outs are detected with the same excitation, detection and dyes in the same liposomes. So in summary, it is possible to determine simultaneously the percentage of docked/fused and free liposomes via FCCS and the percentage of free/docked and fused liposomes via FRET. The combination of both read outs allow for determining the individual percentages of free, docked and fused liposomes. In addition, the auto correlational analysis of the individual channels (i.e. the signals from red, green or other fluorescence tags) allows for determining the relative concentration of the correspondingly tagged liposomes. The assay allows, for example, to determine the progression of free liposomes through docked intermediates to liposomes fully fused by neuronal SNARE proteins (Fig. 2j, k). The direct comparison of the population of the double tagged fused and docked liposomes detected via FCCS (red in Fig. 2j) with that of the fused population detected via FRET (black in Fig. 2j) allows for determining the temporal evolution of docked intermediates (Fig. 2k). This approach was applied to many different combinations of model liposomes, varying in lipid composition, proteins, their mutants and DNA constructs reconstructed into the membranes, liposome sizes and curvatures, as well as liposome environment such as Ca^2+^-concentration or ionic strength (Hernandez et al. 2012; Hubrich et al. 2018; Park et al. 2015, 2014; Vennekate et al. 2012; Yavuz et al. 2018). Thus, this assay constitutes a robust and reliable tool for measuring these fusion intermediates under various conditions.

## Asymmetric labelling of inner and outer membrane leaflets for hemifusion studies

While the previous section addressed the quantification of free, docked/tethered and fused liposomes, we have yet to consider hemifused intermediates. Hemifused liposomes are characterized by a merger between the outer membrane leaflets while the inner leaflets are still separated. In principle, hemifused liposome populations can also be detected with the same FCCS and FRET parameters discussed in the previous section. Theoretically, a sample containing 100% hemifused liposomes would be detected by 100% cross-correlation in FCCS, as all diffusing particles are double tagged, and 50% lipid mixing in FRET, as only half of the membranes are mixed. However, signatures of hemifused liposomes barely were observable so far in two-photon FCCS/FRET studies. Likely this is due to a short lifetime of hemifused intermediates, corresponding to only small populations of hemifused intermediates (Fix et al. 2004; Yoon et al. 2006).

**Fig. 3:**
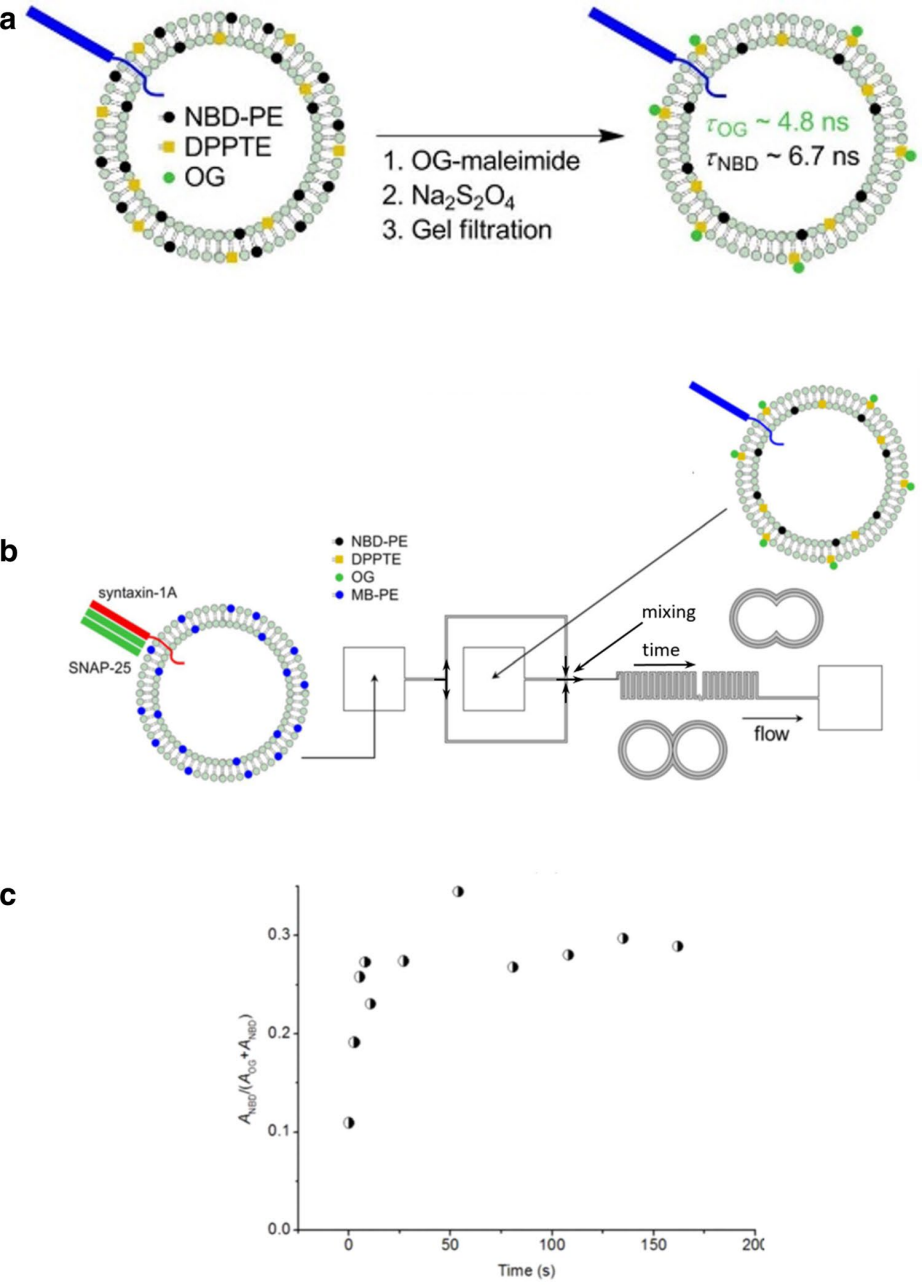
Observation of short-lived hemifused liposomes by asymmetric lipid leaflet labelling. **a** Reaction scheme for asymmetric labelling of the acceptor liposomes including the neuronal SNARE Synaptobrevin-2 (blue). Inner and outer lipid leaflets contain lipid anchored fluorescent NBD (black *filled circle*) and non flluorescent 1,2-Dipalmitoyl-*sn*-Glycero-3-Phosphothioethanol (DPPTE, yellow *filled square*). In a first step, OG-maleimide binds to DPPTE labelling only the outer leaflet. In the second step, sodium dithionite degrades the NBD fluorophores on the outer leaflet. Unbound fluorophores and remaining reactants are removed by gel filtration in a third step. **b** Scheme of the microfluidic channel system to observe short-lived hemifused liposomes. MB-labelled donor liposomes containing the neuronal SNARE partners syntaxin-1A and SNAP-25 are injected into a microfluidic device (left square) and is pumped through the channels as indicated by the arrows. Acceptor liposomes, prepared as described in (**a**), are injected (middle square) and mix with donor liposomes. Close to the mixing spot, liposomes are measured. Continuous pumping through the channel system allowed for elongated observation shortly after mixing. To measure different times, measurement spots far away from the mixing spot are chosen. **c** Relative contribution of the inner leaflet NBD-fluorescence to the measured fluorescence decay. The fast inrease of the inner leaflet NBD-contribution reflects the fast transition from hemi-fused membranes to fully fused membranes. Reprinted with permission from (Lin et al. 2016). Copyright 2020 American Chemical Society

An approach to gain a more direct insight into the presence of hemifused intermediates is asymmetric labelling of the inner and outer membrane leaflets. There are different protocols to achieve asymmetric labelling of the inner and outer membrane leaflets. Here, we will focus on a procedure that is based on selective chemical degradation of the fluorescence tags attached to the lipids of the outer leaflet and subsequent retagging with a different label thereafter (Fig. 3a, black and green dots) (Stengel et al. 2007). Then, the two different tags allow, in general, to dissect membrane fusion of the outer and inner leaflet when detecting the corresponding lipid mixing in the inner and outer leaflet by FRET.

In principle, three possibilities exist for this approach: (1) The outer and inner leaflets of both populations of liposomes are each labelled with completely different FRET donor/acceptor dye pairs. Then, hemifusion is indicated when fluorescence is detected only from the outer leaflet acceptors after spectrally selective excitation of the outer leaflet donors. Full fusion is indicated when additionally, fluorescence is detected from the inner leaflet acceptors after spectrally selective excitation of the inner leaflet donors. Obviously, such a scheme requires very careful selection of spectral ranges for these four different dyes to enable selective excitation of only the outer or inner leaflet donor dye as well as selective detection of only the outer or inner leaflet acceptor dye. (2) Only the donor liposomes contain different tags in the outer and inner leaflet while the acceptor liposomes carry only one tag in both leaflets that can accept energy from both donors. The readout is then the fluorescence lifetime of the different donors. (3) Only the acceptor liposomes contain different tags in the outer and inner leaflet while the donor liposomes carry only one tag in both leaflets that can donate energy to both acceptors. The different fluorescence intensities of the acceptors can be used to distinguish between hemifusion and fusion.

However, when the tags are excited in liposomes with different donors in the outer and inner leaflet, it is difficult to avoid energy transfer from the donor tags with the higher excitation energy to the donor tag with the lower energy before any of the donors encounter any acceptor tag (Lin et al. 2016). In principle, the same problem exists if the acceptor liposomes contain different tags in the outer and inner leaflet. To solve this principle problem, one option is using donor/acceptor dyes for both leaflets with rather similar emission and absorption spectral ranges but with different fluorescence decay times. The fraction of transfer to the acceptor of one or the other can then be separated by measuring the fluorescence decay profile of both acceptors with one single detector but with subsequent biexponential decay fitting with two fixed decay times previously known from the outer and inner leaflet acceptors. The two decay amplitudes derived from this biexponential fit are then proportional to the fraction of energy transfer either within the outer or inner leaflet tag and thus proportional to the outer or inner leaflet fusion.

This approach was realized with Marina Blue (MB) as donor tag in both leaflets of one liposome population and nitro-benzoxadiazole (NBD) as well as Oregon green (OG) as tags in the inner and outer leaflet the other liposome population (Fig. 3b). The acceptor tag lifetimes were with 6.7 ns and 4.8 ns, respectively, quite different and provided, therefore, a good dynamic range for observing transitions from transfer only to the outer leaflet (hemifused states) to transfer to both leaflets (full fusion) (Lin et al. 2016).

This assay is much more sensitive for hemifused intermediates than the FCCS/FRET of the previous section. With this assay it was possible to observe hemifused liposomes for about 5 s after mixing in a microfluidic channel system (Fig. 3b). Directly after mixing, the detected acceptor fluorescence decay was very similar to the decay of the outer leaflet tag (OG, ~ 4.8 ns). However, this decay increased rapidly after mixing to a value approximately between the decay values of the outer and inner leaflet tag (NBD, ~ 6.7 s) and remained constant after approximately 5 s (Fig. 3c). This very short lifetime of the hemifused intermediates explains why the population of these intermediates are hard to detect without an assay particularly sensitive for hemifusion.

## Determining membrane distances below 10 nm using transmembrane energy transfer and calibration with fixed DNA structures

**Fig. 4:**
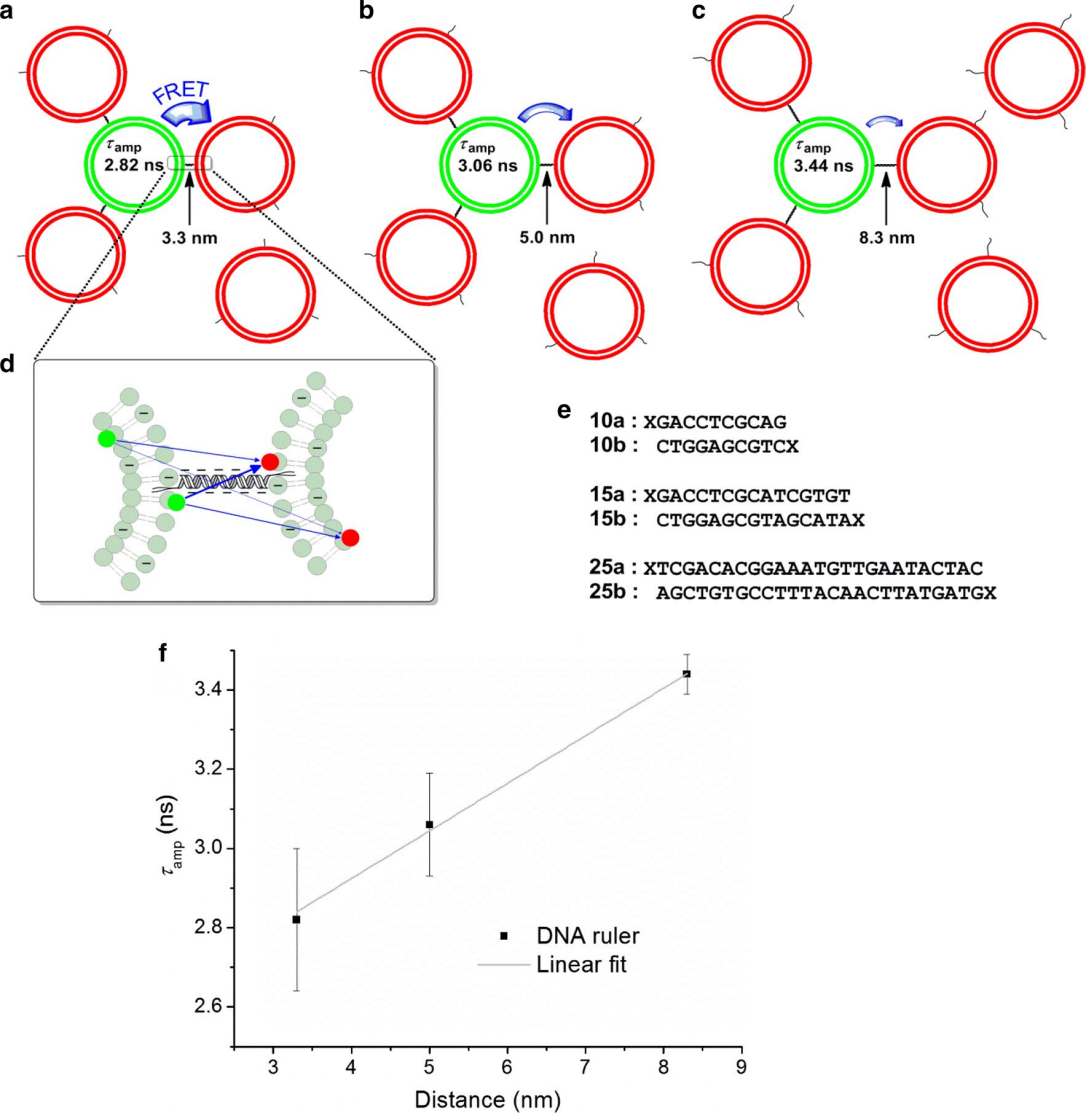
Calibration of the membrane distance ruler. **a**–**c** Principle of the membrane distance calibration with lipid anchored DNA. Donor liposomes (green) are surrounded by an excess of acceptor liposomes (red). The distance between both membranes is set by the length of the DNA pairs and resulted in different FRET efficiencies. **d** Each donor fluorophore (green) is in proximity of multiple acceptor fluorophores (red). The closer they are, the more efficient is the intermembrane energy transfer. **e** DNA-sequences used to set the membrane distances shown in (**a**–**c**). X represents the lipid anchor. **f** Membrane distance calibration plot. The average fluorescence lifetime *τ*_amp_ is plotted against the known membrane distance set by the used DNA pair and fitted with a linear model. Reprinted with permission from (Lin et al. 2014)

During the exploration of membrane interactions in neuronal signal transmission, it became apparent that the regulation of membrane distances at a sub 10 nm scale might be an important factor governing the triggering and/or transmission mechanism (van den Bogaart et al. 2011). A classic approach to determine distances and their fluctuations on such a scale in biological systems is tagging two points to be measured by a donor and acceptor dye, respectively, and calculate their distance using Försters theory for energy transfer (Brunger et al. 2009; Choi et al. 2010). However, in contrast to determining the distance between two proteins in a defined protein complex or between two side-chain positions in a stably folded individual protein, a complex distribution of different donor–acceptor distances  is present when membranes contain labelled lipids that can diffuse in the membrane (Fig. 4d). In principle, the average intermembrane energy transfer could be computed by advanced theoretical modelling but a more direct approach is to calibrate the observed donor fluorescence decay time as a function of intermembrane distance. To realize such a calibration, we decided to use membrane anchored synthetic DNA of exactly predetermined lengths (Fig. 4a–c, e) (Chung et al. 2013). Using DNA of different length allows to generate a calibration plot of donor fluorescence lifetimes corresponding to different intermembrane distances (Fig. 4f). Please note that a sufficient change in the donor lifetime was achieved here using multiple acceptor liposomes surrounding an individual donor liposome. Otherwise, the change would be much smaller as in the case of docked/tethered 1:1 liposomes described earlier in this paper and significantly smaller than observed for the intramembrane energy transfer of fused liposomes. Using multiple acceptor liposomes of high concentration around individual donor liposomes ensures that a large fraction of the donor liposomes surface is in close proximity to acceptors, which leads to large changes in the fluorescence decay of the donor.

**Fig. 5:**
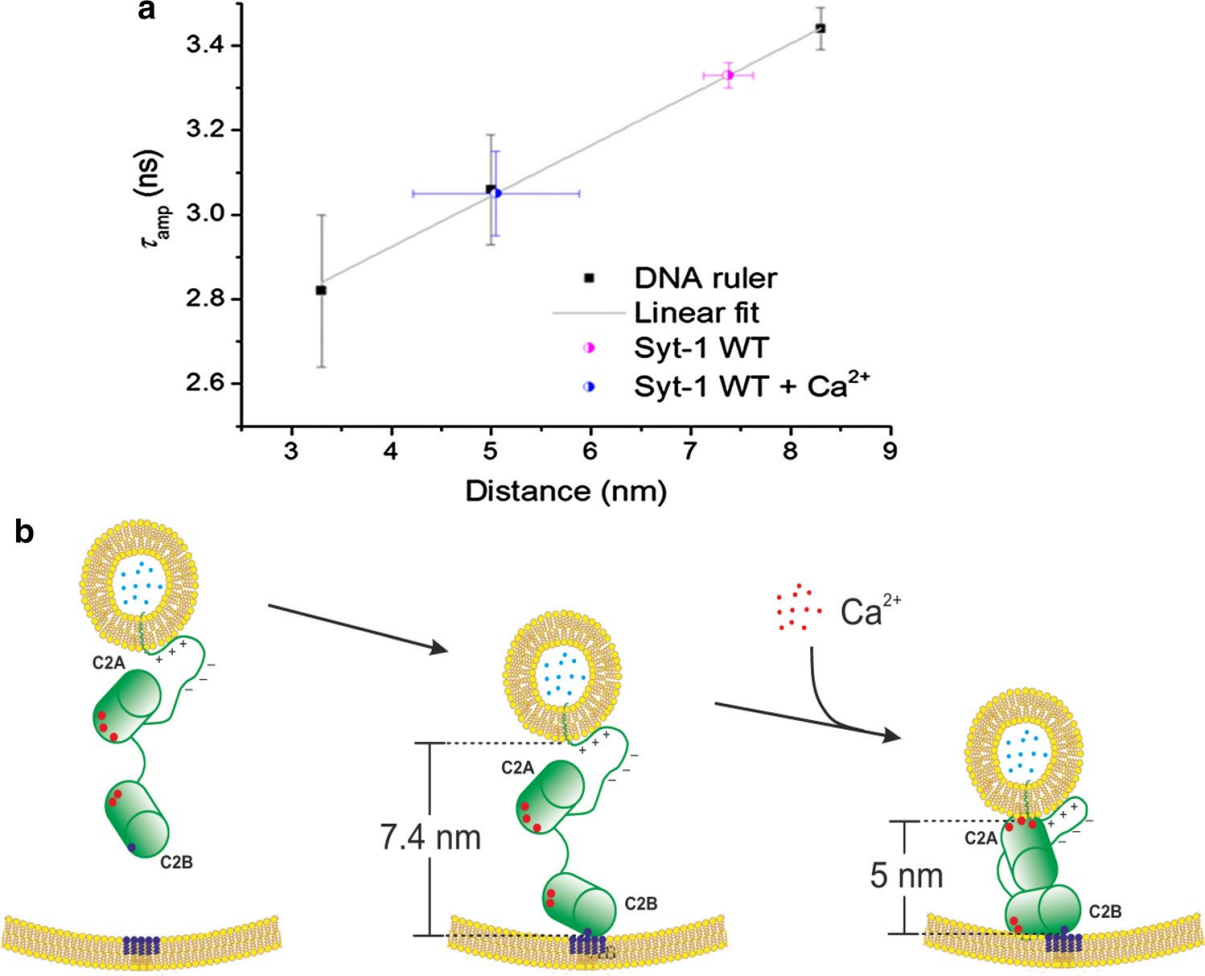
Synaptotagmin 1 crosslinks two membranes and reduces their distance upon Ca^2+^-triggering. **a** The membrane distance calibration plot as shown in Fig. 4f, added by the observed lifetime of wild-type Synaptotagmin 1 in the absence (pink partially filled circle) or presence (blue partially filled circle) of 100 µM Ca^2+^. **b** Model of how Synaptotagmin 1 regulates the membrane distance. Without Ca^2+^, Synaptotagmin 1 binds to phosphatidylinositol 4,5-bisphosphate in the presynaptic membrane. The distance between both membranes is held at 7–8 nm. On Ca^2+^ influx, its C2 domains bind to the opposing membranes and reduce their distance to 5 nm. Reprinted with permission from (Lin et al. 2014)

Using the calibrated membrane distance ruler allowed, for example, to determine the distances between liposomes that are clustered by synaptotagmin 1 in the presence and absence of physiological concentrations of 100 µM Ca^2+^ (Fig. 5). Synaptotagmin 1 triggers the fusion of synaptic vesicles with the presynaptic membrane when the Ca^2+^ concentration increases to values of about 100 µM (Park et al. 2015). Experiments using liposomes reconstituted only with synaptotagmin 1 demonstrated that the membrane distances decreased from about 7.5 nm (pink in Fig. 5a) in the absence of Ca^2+^ to about 5 nm in the presence of 100 µM Ca^2+^ (blue in Fig. 5a). While the physiological processes are certainly much more complex, such experiments demonstrate that synaptotagmin 1 can—in principle—regulate membrane distances as a function of physiological Ca^2+^ concentrations (Fig. 5b).

## Summary

Complex interactions between membranes and proteins are governing a major fraction of decisive processes in the biosphere but are hard to dissect on a molecular level. Certainly, in living organisms, these processes are more than just the sum of individual molecular mechanistic steps. However, dissecting interactions on an individual protein/membrane level in a highly controlled and well defined manner is still the prerequisite for figuring out the key elementary steps that form the basis for the understanding of the whole system. Model systems in which liposomes freely diffuse in aqueous solutions are well suited to observe these key elementary steps of membrane interactions. It is a demanding task establishing liposome models that are very well defined with respect to lipid composition of inner and outer leaflets, membrane curvature and tension, reconstitution of wild-type and mutant proteins governing these interactions into the membranes, properties of the intra- and extra-liposomal space as well as tagging of distinct membrane parts or proteins. An unbiased determination of the membrane interactions is an important challenge that is best addressed using advanced biophysical methods. Combining different multiphoton-excitation, fluorescence correlation, FRET and fluorescence lifetime approaches in confocal microscope set-ups provides a multitude of valuable and direct insights into these individual inter membrane and protein key interactions. Dual-colour two-photon FCCS together with time-resolved intramembrane FRET allows robustly dissecting transformations between free, docked/tethered and fused membranes and determining how specific proteins influence these parameters. Asymmetric labelling of inner and outer membrane leaflets combined with time-resolved double FRET and microfluidic channel mixing allows observation of hemifused membranes preceding full fusion. Time-resolved intermembrane FRET allows for observation of membrane distance regulation by proteins like synaptotagmin 1 and its regulation by Ca^2+^.These techniques thus provide valuable insights into intermediates that are difficult to characterize by other approaches, for instance docked intermediates preceding synaptic SNARE protein driven membrane fusion, hemifusion states occurring before full fusion or the regulation of membrane distances by the neuronal calcium sensor synaptotagmin on a sub 10 nm scale when the Ca^2+^ concentration is varied within physiological limits. We expect that the future will provide even more exciting insights from these techniques that go beyond membrane/protein interactions important in neuronal signal transmission.
